# Malignant Extrarenal Rhabdoid tumor derived from the greater omentum: A case report and literature review

**DOI:** 10.1002/cnr2.2086

**Published:** 2024-05-20

**Authors:** Xue Shao, Meijun Liu, Xin Wang, Lingling Han, Shuang Luo

**Affiliations:** ^1^ School of Medical and Life Science Chengdu University of Traditional Chinese Medicine Chengdu China; ^2^ Department of Gynecology Suining Central Hospital Suining China

**Keywords:** acute abdomen, chemotherapy, extrarenal malignant rhabdoid tumor, greater omentum

## Abstract

**Background:**

Malignant extrarenal rhabdoid tumor (MERT) is a rare and highly metastatic tumor, which is more than 75% of patients dying within 6 months of initial diagnosis, and it often leads to misdiagnosis and delayed treatment.

**Case:**

This paper reports a 16‐year‐old girl who presented with the chief complaint of acute abdominal pain. She underwent laparoscopic exploration and excisional biopsy, then pathological examination and immunohistochemistry revealed “extrarenal malignant rhabdomyoma.” One month after operation, she died of intra‐abdominal hemorrhage and multiple organ dysfunction.

**Conclusion:**

MERT were often misdiagnosed and had a poor prognosis. The surgery and chemotherapy are usually beneficial to prolong the survival time of patients with MERT.

## 1 INTRODUCTION

Malignant rhabdoid tumor (MRT) is a highly aggressive malignant tumor, which was first discovered in the kidneys of infants by members of the Wilms tumor team in 1978.^1^ It was subsequently reported in various extra‐renal sites including central nervous system, stomach, colon, liver, bladder, uterus, ovary, and soft tissues,^2^, ^3^ which are called malignant extrarenal rhabdoid tumor (MERT). Because of MERT rapid growth and spreading, these patients usually have a relatively short (in the order of weeks, or even days) clinical presentation characterized by the onset of progressive symptoms. Clinical symptoms are related to the site of the tumor. The diagnostic work‐up includes computerized scanning examination; however, MERT cannot be reliably distinguished from other diseases without histopathological examination, therefore, surgery is necessary to obtain tissue and confirm the diagnosis. Histologically, the tumor cells had eccentric nuclei and prominent nucleoli.^4^ The cytoplasm was eosinophilic and often contained clear inclusions that replaced the nucleus. On immunohistochemical staining, these cells were usually positive for vimentin and epithelial markers. Cytogenetic studies have shown that classic MERT contain a characteristic chromosomal abnormality involving chromosome 22.^5^, ^6^

We reviewed 14 cases of MERT admitted with “acute abdominal pain” in the past decade (Table 2) and found that there was no unified expert consensus for the diagnosis, and it often leads to misdiagnosis and delayed treatment, in order to mainly explore the clinical diagnosis and treatment of MERT, improving the understanding of MERT with abdominal pain as the main manifestation. We report the first case of MERT derived from the greater omentum, which was admitted with acute abdomen.

## 2 CASE REPORT

**FIGURE 1:**
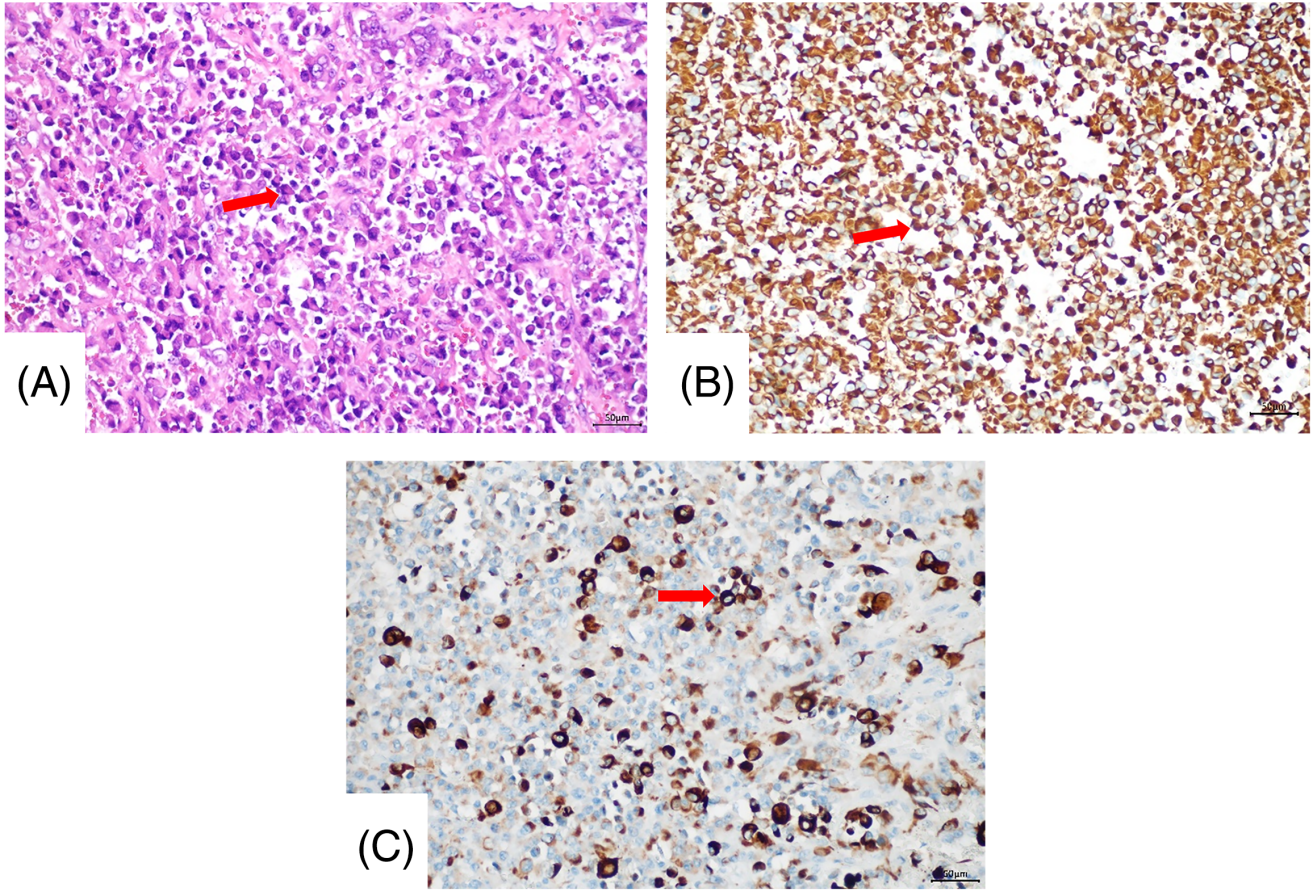
(A) Typical rhabdoid cells with eosinophilic intracytoplasmic inclusions, and eccentric nuclei with prominent nucleoli (H and E, ×200) (arrow). (B) vimentin stain showing cytoplasmic positivity (×200) (arrow). (C) Cytokeratin stain showing cytoplasmic positivity (×200) (arrow).

**FIGURE 2:**
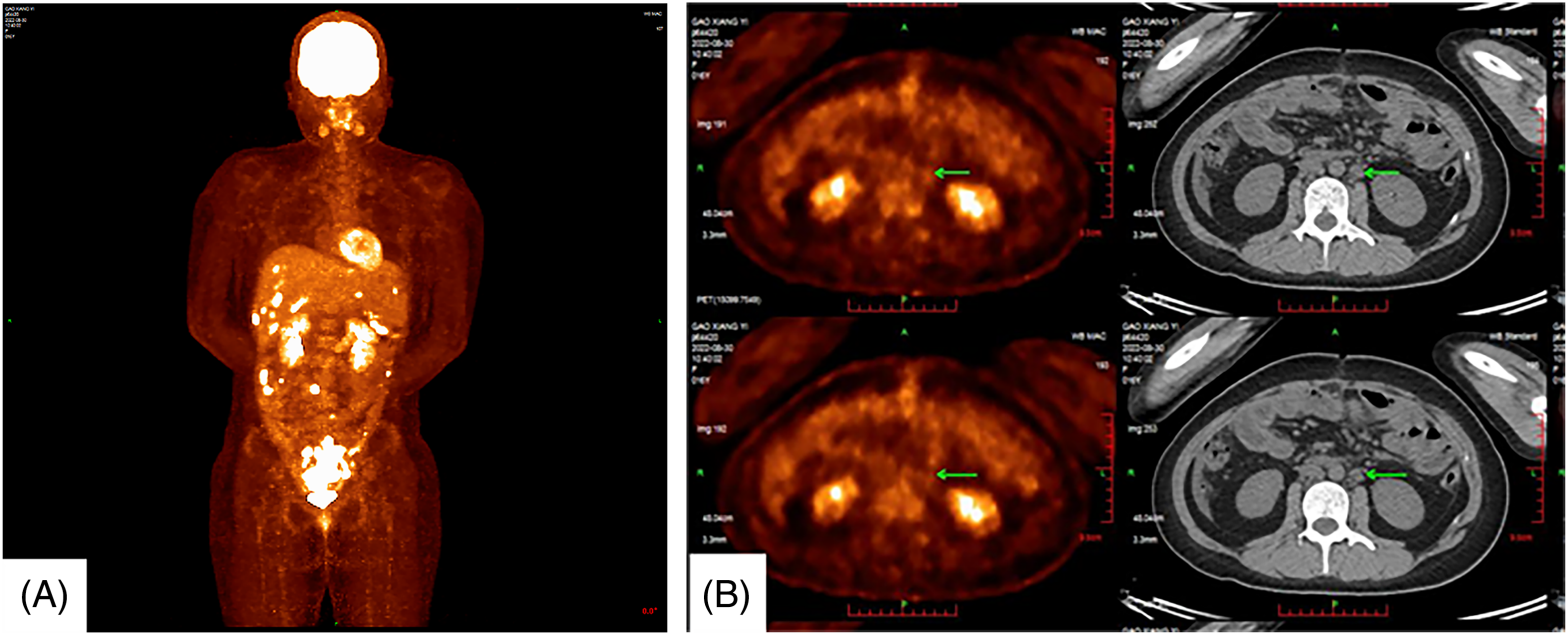
(A) Abnormal increase of glucose metabolism in multiple nodules and masses in the abdomen and pelvis, and multiple peritoneal metastasis of multiple tumors. (B) Multiple abdominal lymph nodes were shown, some of which were enlarged, accompanied by a slight increase in glucose metabolism, the largest located at the level of the lower part of the left kidney near the abdominal aorta. (arrow).

The patient was 16 years old, and was admitted to our center, Suining Central Hospital, with acute abdominal pain for more than 10 days on August 16, 2022. Ten days ago, she developed severe paroxysmal abdominal pain without an obvious trigger, which was relieved after rest, and without any other discomfort. Half a day ago, she developed worsening abdominal pain without obvious inducement, accompanied by marked nausea and vomiting. Ultrasound showed that a cystic about 13 cm × 11 cm × 15 cm was found in front of the uterus, which was considered to be ovarian tumor pedicle torsion. Routine blood tests were normal and serum tumor markers were CA125:1033 U/mL (The normal range for CA125 is less than 47 U/mL), and the rest are within the normal range. Based on the patient's clinical symptoms and clinical experience, the patient chose emergency laparoscopic exploration on the day of admission. Operative notes illustrated that dense adhesion of the greater omentum and intestine formed a fish‐like mass with a size of about 15 cm × 15.0 cm, with rupture, hemorrhage, and necrosis. Intraoperatively, there was approximately 1000 mL hemorrhage in the abdominal cavity. Many scattered purplish red nodules between 1.0 cm × 0.8 cm and 4.0 cm × 4.5 cm were seen in the greater omentum. Because the nature of the mass could not be determined during the operation, only omentectomy and ovarian biopsy were performed after communication with the patient's family. The pathological results of ovarian tissue from both sides showed no tumor. Postoperative pathological examination of greater omentum showed that it was a malignant tumor. Histologically, the neoplastic cells showed large, vesicular, and eccentric nuclei with conspicuous eosinophilic nucleoli and abundant cytoplasm (Figure 1A). And immunohistochemistry showed that vimention and CK (AE1/AE3) were positive (Figure 1B,C), and Bcl‐2, CD34, CK20, SMA, and WT1 (Wilms' tumor) were negative. Further detection of INI‐1 was also negative. Finally, the pathological diagnosis of the greater omental mass was MERT. Unfortunately, the patient refused further treated with surgery and chemotherapy. The postoperative period was uneventful and she was discharged from the surgical unit on postoperative day 8. Then, she went to West China Hospital for PET/CT, and the results showed: (1) abnormal increase of glucose metabolism in multiple nodules and masses in the abdomen and pelvis, and multiple peritoneal metastasis of multiple tumors (Figure 2A). (2) Multiple abdominal lymph nodes were shown, some of which were enlarged, accompanied by a slight increase in glucose metabolism, the largest located at the level of the lower part of the left kidney near the abdominal aorta (Figure 2B). Finally, she died of intra‐abdominal hemorrhage and multiple organ dysfunction 1 month after operation (Table 1).

**TABLE 1:** Clinical, diagnostic, and therapeutic chronological timeline.

09 August 2023	Ultrasound (Pelvic mass about 13 cm × 10 cm)
16 August 2023	Ultrasound (a cystic about 13 cm × 11 cm × 15 cm was found in front of the uterus); Serum tumor markers (CA125:1033 U/mL, and the rest are within the normal range)
16 August 2023	Preliminary diagnosis (ovarian tumor pedicle torsion)
16 August 2023	Emergency laparoscopic exploration (omentectomy and ovarian biopsy)
17 August 2023	Ultrasound (Ascites and some intestinal pneumatosis)
23 August 2023	IHC (CK (AE1/AE3) and vimentin were positive, and Bcl‐2, CD34, CK20, SMA, WT1 (Wilms' tumor), and INI‐1 were negative)
24 August 2023	Discharge from hospital
30 August 2023	PET‐CT (abnormal increase of glucose metabolism in multiple nodules and masses in the abdomen and pelvis, and multiple peritoneal metastasis of multiple tumors)
One month after operation	Died of intra‐abdominal hemorrhage and multiple organ dysfunction

## 3 DISCUSSION

MRT was originally described as a rare renal sarcoma of infancy and early childhood, but the tumors with a similar histological and clinical appearance, either partially or globally, have been reported in various extrarenal sites,^3^ such as the soft tissue, brain, heart, liver, pancreas, uterus, bladder, prostate, stomach, skin, and colon.^7^ We herein reported a very rare case of a MRT, which probably originated in the greater omentum. As far as we could determine in the past literature, no case originating in the greater omentum has been reported.

We performed a systematic review of the literature regarding cases of MERT admitted with “acute abdominal pain” in recent 10 years (Table 2). Analysis of these cases revealed that age at presentation was 40–76 years, and the primary lesions were all located in the digestive system. Only two cases were considered to be cancer before surgery and the rest were usually diagnosed as common acute abdomen. The misdiagnosis rate was 75%. Their prognosis is very poor and over 75% of the patients die within 6 months following the initial diagnosis.^12^ Historically, MERT of the digestive system reported in the literature primarily affect older people, with no gender preference. These reports on tumor markers in MERT are almost negative, and the clinical manifestations are not specific, which makes it difficult to be associated with malignant tumors and leads to misdiagnosis. Most reported cases revealed a solid mass with a necrotic center by computed tomography.^15^ Our case is a female adolescent admitted to hospital with acute abdomen as the main complaint. Ultrasound revealed pelvic mass and only CA125 was elevated, among the tumor markers, which was preliminarily considered as ovarian cyst torsion based on clinical experience. For young women, with rapid onset and no clear tumor indicators abnormal, it is difficult to consider malignant tumor. According to the clinical guidelines, emergency laparoscopic exploration was performed, and the lesions were scattered on the omentum. The nature of the tumor could not be determined during the operation and lack of experience, only an epiploectomy and ovarian biopsy were performed. For rare diseases, tissue should be obtained as early as possible to confirm the diagnosis. And fine‐needle aspiration cytology is an effective method for making a preoperative diagnosis of MERT.^16^

**TABLE 2:** Reported cases of admitted to hospital with abdominal pain in the last decade.

References	Age/sex	Primary site	Preoperative diagnosis	Diagnosis method	Metastasis	INI‐1	Laboratory examination		Tumor marker	Treatment	Prognosis
							positive	negative			
Haikun Ye et al.^8^	40/F	Liver	Liver‐occupying lesion, HCC rupture hemorrhage?	Immunohistochemistry	Abdomen and pelvis	‐	CK 8/18, CD56, SMA, Vim	/	‐	Surgery and Chinese medicine	Alive (24 month)
Cho IJ et al.^9^	73/M	Cecum	Acute appendicitis	Immunohistochemistry	Lymph node, liver and bone	/	CK, VMT, MLH‐1	SMA	/	Surgery	Died (2 month)
D'Amico F et al.^10^	65/M	Colon	No preoperative diagnosis	Immunohistochemistry	Lymph node	‐	Vim, EMA	CK7, CD34/31/20/3/45, C‐Kit, OCT‐2	‐	Surgery	Alive (48 month)
Voglino C et al.^11^	76/M	Small intestine	No preoperative diagnosis	Immunohistochemistry	Lymph nodes	‐	CK, Vim	CD20/3/117, HMB45, S100	‐	Surgery	Dide (2 month)
Öfkeli Ö et al.^12^	70/F	Stomach	Gastric carcinoma	Immunohistochemistry	Lymph nodes and liver	/	Vim	/	‐	Surgery and chemotherapy	/
Xu QH et al.^13^	67/M	Spleen	Rupture of splenic tumor with subcapsular hemorrhage of spleen	Immunohistochemistry	Liver, lungs and bones	/	CK, EMA, Vim	/	/	Surgery	Recurrence with metastasis (2 month)
Parker NA et al.^14^	56/M	Retroperitoneum	No preoperative diagnosis	Immunohistochemistry	No distant metastasis	‐	CD34, EMA	CK AE1/AE3, S100, MSA	/	Surgery and neoadjuvant chemotherapy	Alive(18 month)

*Note*: (−) negative; (/) not mentioned.

Abbreviations: F, female; HCC, hepatocellular carcinoma; M, male.

The exact nature of which could only be verified following thorough immunohistochemical analysis.^16^ Histologically, the tumor cells are highly cellular, arranged in solid sheets or in alveolar or trabecular patterns with abundant eosinophilic cytoplasm containing globular hyaline‐like inclusions, and resemble rhabdomyoblasts.^17^, ^18^ Immunohistochemical staining for vimentin, cytokeratin, and cytoplasmic epithelial membrane antigen has generally revealed positive results in MERT.^16^, ^19^, ^20^ Our case showed similar histological findings under hematoxylin‐eosin staining. Cytogenetic and molecular analysis revealed a molecule‐analyzed abnormality of the long arm of chromosome 22 (22q11.2), which encodes the INI‐1 gene, a core subunit of the SWI/SNF ATP‐dependent chromatin remodeling complex.^21^ INI‐1 is widely expressed in the nuclei of all normal cells. According to the literature, several experimental studies have shown that INI‐1 heterozygous deficient mice develop undifferentiated sarcomas with rhabdoid cytological features, suggesting that INI‐1 is an important tumor suppressor gene.^6^ The immunohistochemical examination of the patient reported here also confirmed whether INI‐1 loss indicates that the mechanism of this disease is caused by abnormal gene expression.

For differential diagnosis, histologic and IHC findings should be kept in mind and Wilms' tumor, GIST, sarcoma, malignant melanoma, and lymphoma should be considered.^22^, ^23^, ^24^ Because of the aggressive clinical behavior and poor prognosis of the disease, its differential diagnosis should be made, however, at present, the differential diagnosis is a great challenge, which is worthy of further exploration.

All patients underwent surgical resection as the first line of therapy. The rare occurrence of MERT has made it complicated to establish adequate survival‐improving protocols. Francesco D'Amico et al. suggested that there was a correlation between performing an R0 resection in the first place and prognosis.^10^ It has also been proposed that radiological evaluation should be performed to assess locoregional and distant spread of the tumor prior to treatment planning,^25^ but the effect of surgical treatment on prognosis and survival of patients was limited. Parker et al. reported a case of MERT admitted to hospital with acute abdomen, who underwent the surgery and postoperative chemotherapy and obtained the survival period of 18 months.^14^ While the case we reported was confirmed by pathology after MERT, due to the rarity of the disease and lack of clinical experience, and she is an adolescent girl, our team was unable to implement an appropriate treatment plan. Finally, the patient chose to be discharged from the hospital without further surgery and chemoradiotherapy. Her condition progressed rapidly after discharge, and PET‐CT shows abnormal increase of glucose metabolism in multiple nodules and masses in the abdomen and pelvis, died of intra‐abdominal hemorrhage and multiple organ dysfunction 1 month after operation. Through reviewing MERT cases with chemotherapy in the recent 10 years (Table 3), it can be seen that chemotherapy is beneficial to prolong survival and bring more opportunities for surgery. VIDE chemotherapy regimen has some potential benefits for the survival of patients, and Wolfe et al. have reported two patients with neck MRT obtained longer survival with aggressive multimodal alkylator‐based chemotherapy, local control with XRT, and consolidative high dose chemotherapy with autologous stem cell rescue (HDC‐ASCR) using CEM conditioning,^28^ but more reports and studies are needed to confirm it. However, the current curative effect of radiotherapy is not exact. Yasui et al. reported a case in which the lesion was improved by preoperative radiotherapy and then relapsed resulting in exacerbation of the disease.^27^

**TABLE 3:** Reported cases of MERT treated with chemotherapy in recent 10 years based on literature survey.

Author	Age/sex	Primary site	Metastasis	Treatment	Chemotherapy regimens	Side effects	Efficacy	Prognosis
Asada N et al.^26^	7‐year‐old/F	Left cubital fossa	No metastasis	Preoperative chemotherapy, surgery, and postoperative chemotherapy	VDC + ICE	Hematological toxicity	Reduction in tumor volume, disappearance of FDG uptake	Alive (2 year)
Parker et al.^14^	56‐year‐old/M	Left upper abdominal pain	No metastasis	Surgery and neoadjuvant chemotherapy	VIDM	Neutropenic fever and sepsis	PET/CT showed complete resolution of the tumor	Alive (18 month)
Yasui et al.^27^	2‐year‐old/F	Left scapula	Lung	Chemotherapy	VIDE	Hematological toxicity and febrile neutropenia	The primary and primary lesions disappeared	Alive (31 month//27 months after VIDE)
	3‐year‐old/F	the fifth thoracic vertebra	No metastasis	Surgery, and postoperative chemotherapy	TE for one cycle, CV for two cycles; VIDE for six cycles	Hematological toxicity	TE + CV: aggravation of illness VIDE: stable condition	Alive(33 month//13 months after VIDE)
	7‐year‐old/F	Left thigh	/	Preoperative chemotherapy, surgery, and postoperative chemotherapy	VAC + VIDE	Hematological toxicity	VAC: The primary tumor continued to grow; VIDE: Reduction in tumor volume	Alive (23 month/17 months after VIDE)
Wolfe et al.^28^	13‐year‐old/F	Neck	No metastasis	Chemotherapy + Radiation therapy	VDCCE + HDC‐ASCR utilizing CEM conditioning	Sequelae of radiotherapy	Stable condition	Alive (7 year)
	19‐month‐old/F	Neck	No metastasis					Alive (53 month)
Szymanski et.^29^	9‐year‐old/M	Urinary bladder	No metastasis	Preoperative chemotherapy, surgery, and postoperative chemotherapy and radiation therapy	VDC + CCE	/	Stable condition	Alive (6 month)

*Note*: (/) not mentioned.

Abbreviations: CCE, carboplatin, cyclophosphamide, and etoposide; F, female; ICE, ifosfamide, carboplatin, and etoposide; M male; TE, temozolomide and etoposide; VAC, vincristine, dactinomycin, and cyclophosphamide; VC, vinorelbine and cyclophosphamide; VDC, vincristine, doxorubicin, and cyclophosphamide; VDCCEC, vincristine, doxorubicin, cyclophosphamide, carboplatin, and etoposide; VIDE, vincristine (VCR), ifosfamide (IFO), doxorubicin (DOX), and etoposide (VP); VIDM, vincristine, doxorubicin, ifosfamide, and mesna.

## 4 CONCLUSION

MERT are characterized by rare, high malignancy, and poor prognosis. We herein reported the first case of MERT, which probably originated in the greater omentum. The misdiagnosis rate of MERT admitted with acute abdomen is high and the prognosis is poor. For young female patients with abdominal pain as the main complaint, the possibility of malignant tumor should be considered after the common acute abdomen is excluded, and the progression rate of the disease should be vigilant. Our case was misdiagnosed as ovarian tumor pedicle torsion before surgery due to the lack of specific clinical manifestations and ultrasonography and lack of clinical experience. After diagnosis, there was no supplementary operation and chemoradiotherapy, resulting in rapid progression of the patient's condition and finally death 2 months after surgery. In conclusion, to obtain pathology results as soon as possible to determine the nature is of paramount importance, and screening for INI‐1 status is also an important clue for diagnosis and future therapeutic directions. However, the rarity of the disease has made it difficult to determine adequate protocols. Performing an R0 resection and chemotherapy intervention is beneficial to improve the prognosis, and the use of radiotherapy did not improve survival in adults with MERT.^30^

## AUTHOR CONTRIBUTIONS

**Xue Shao:** Writing – original draft; writing – review and editing; conceptualization; investigation; data curation. **Meijun Liu:** Data curation; conceptualization; investigation; writing – original draft. **Xin Wang:** Writing – review and editing. **Lingling Han:** Conceptualization. **Shuang Luo:** Writing – review and editing; supervision; data curation; conceptualization.

## CONFLICT OF INTEREST STATEMENT

The authors have stated explicitly that there are no conflicts of interest in connection with this article.

## ETHICS STATEMENT

We obtained a written statement of informed consent from the patient for the publication of case details and the use of images. The case discussed in this manuscript does not include patient‐identifying information.

## Data Availability

The data that support the findings of this study are openly available in pubmed at https://pubmed.ncbi.nlm.nih.gov/.
